# Alteration of Political Belief by Non-invasive Brain Stimulation

**DOI:** 10.3389/fnhum.2015.00621

**Published:** 2016-01-21

**Authors:** Caroline Chawke, Ryota Kanai

**Affiliations:** ^1^School of Psychology, University of SussexBrighton, UK; ^2^Sackler Centre for Consciousness ScienceBrighton, UK; ^3^Department of Neuroinformatics, Araya Brain ImagingTokyo, Japan

**Keywords:** transcranial random noise stimulation (tRNS), dorsolateral prefrontal cortex (DLPFC), political neuroscience, cognitive dissonance, goal directed reasoning, unconscious processing, belief formation

## Abstract

People generally have imperfect introspective access to the mechanisms underlying their political beliefs, yet can confidently communicate the reasoning that goes into their decision making process. An innate desire for certainty and security in ones beliefs may play an important and somewhat automatic role in motivating the maintenance or rejection of partisan support. The aim of the current study was to clarify the role of the DLPFC in the alteration of political beliefs. Recent neuroimaging studies have focused on the association between the DLPFC (a region involved in the regulation of cognitive conflict and error feedback processing) and reduced affiliation with opposing political candidates. As such, this study used a method of non-invasive brain simulation (tRNS) to enhance activity of the bilateral DLPFC during the incorporation of political campaign information. These findings indicate a crucial role for this region in political belief formation. However, enhanced activation of DLPFC does not necessarily result in the specific rejection of political beliefs. In contrast to the hypothesis the results appear to indicate a significant increase in conservative values regardless of participant's initial political orientation and the political campaign advertisement they were exposed to.

## Introduction

“*So convenient a thing is it to be a rational creature, since it enables us to find or make reason for everything one has a mind to”*

-Ben Franklin

Ideology has been described as a “powerful motivational force” (Jost and Amodio, 2012), so strongly inspirational that it enables individuals and groups to maintain reason for an abstract configuration of ideas and values, often to the detriment of their own lives. Contemporary politics are characterized by partisanship and polarization, with clearly defined lines separating the ideologies of a liberal or conservative, left- or right- wing orientation. Therefore, it is no surprise that these labels offer a strong sense of social identity and moral contentment toward ones political affiliation (Tajfel and Turner, 1979; Hardin and Higgins, 1996).

However, As Lieberman and Schreiber (2003) point out; in terms of belief formation, particularly with regards to political thinking, people are generally unaware of how little insight they have into their own decision making process. Given that most individuals are capable of orientating to two or more sides of a political debate, the way in which they subsequently appraise political issues may be strongly influenced by the specific feelings aroused during the early stages of processing (Lodge and Taber, 2013). This poses a challenge to the credibility of self-report measures in politics, with increasing need for an investigation into the stability of political beliefs and the neural basis of partisan support.

The intensity and pervasiveness of political belief systems has been recently attributed to the uncertainty-reducing function of ideology (Jost and Amodio, 2012). For instance, researchers have argued that, rather than providing a means of emotional satisfaction *per se*, ideology is motivating because it offers a rationalization mechanism of coping with anxiety and uncertainty, thereby conferring existential security (Hogg, 2007; Jost et al., 2009). Therefore, political beliefs manifest a divergence from purely self-interested decisions, with social categorization demonstrating enhanced activity in reward-related regions of the brain (Fehr and Camerer, 2007; Lee, 2008; Lowenstein et al., 2008).

According to cognitive dissonance theory (Festinger, 1957), the simultaneous processing of two or more conflicting cognitions, results in a psychologically uncomfortable state of arousal. This is thought to initiate a motivational mechanism through which one alters their preferences and beliefs to be more consistent with the displayed behavior. Therefore, executive control mechanisms appear to be involved in the rationalization process whereby the outcome is based on an inhibition of currently suboptimal responses (Harmon-Jones et al., 2008; Mansouri et al., 2009). Studies have suggested an indispensable role of the dorsolateral prefrontal cortex (DLPFC) in this process due to its role in encoding and maintaining short-term memory of experienced conflicts, as well as in adjusting the level of cognitive control according to previous experience (Petrides, 2000; Miller and Cohen, 2001; Tsujimoto and Sawaguchi, 2004, 2005; Genovesio et al., 2006; Mansouri et al., 2007; Weissman et al., 2008). For example, fMRI studies have demonstrated enhanced activity of the bilateral DLPFC during the processing of political judgments which are paired with respectively inconsistent political information (Westen et al., 2006; Kaplan et al., 2007). Similarly, DLPFC has also been implicated during the observation of opposing candidate faces, with greater activity representing reduced affiliation with the opposing candidate as well as their associated political position (Knutson et al., 2006; Westen et al., 2006; Kaplan et al., 2007; Zamboni et al., 2009).

Of particular importance to the current investigation, Kato et al. (2009) provide evidence that distinct prefrontal areas of the brain are associated with different cognitive control mechanisms following exposure to negative representations of their initially favored political candidate. Consistent with earlier studies of emotion biased reasoning (Kunda, 1990; Thagard, 2003; Westen et al., 2005, 2006); persistent support for one's political ideology or candidate, was shown to be associated with activity in both the ventral medial and anterior prefrontal cortices. This research points to the specific role of emotion and reward in the maintenance, as opposed to rejection of political support. In contrast, a negative correlation was found between the activation level of bilateral DLPFC and preference for previously supported candidates following exposure to both negative and positive political campaigns. That is, DLPFC activity was stronger in those who changed their preferences for initially preferred political candidates regardless of the emotional content of the advertisement.

These findings provide support for qualitatively different neural processes underlying goal-directed behavior. Specifically, it points to the role of a deliberative mechanism for the regulation and control of behavior and a more intuitive mechanism that underlies emotionally significant decision making (e.g., Dunbar and Fugelsang, 2005). The specific enhancement of activity in DLPFC during belief alteration has been interpreted as the neural basis for inductive reasoning (Goel and Dolan, 2004; Kato et al., 2009). That is, regardless of the individual's prior beliefs, including the strength of their emotional involvement (Kato et al., 2009), error feedback processing in the DLPFC appears to signal an informative cue that one has made an incorrect decision. In effect, this initiates goal directed action to alter their thinking process when presented with a similar choice in the future (Walton et al., 2004; Zanolie et al., 2008).

From the perspective of social information processing; this could be taken to suggest an inconsistency between experienced and expected social norms. Importantly, research has demonstrated that the detection of non-compliant behavior and its subsequent corrective action depend on the same neurocognitive mechanisms that are known to be involved in goal-directed actions in non-social domains. For example, Klucharev et al. (2009) used a face attractiveness rating task to investigate how subject's ratings were influenced by normative group opinion. They found that a conflict of opinion led to enhanced activation in the posterior medial frontal cortex (pMFC) and a deactivation in reward related regions of the brain (NAc). The pMFC closely interacts with the lateral prefrontal cortex (LPFC), which in turn, implements the necessary top-down control (Kerns et al., 2004; Carter and Van Veen, 2007). This would explain the alteration in beliefs that occurs following increased activation of these regions (Klucharev et al., 2009; Campbell-Meiklejohn et al., 2010). Further, recent research has demonstrated that Cathodal tDCS (negatively charged electrodes) over the lateral prefrontal cortex decreases the expected behavioral adjustments that would typically occur following deviations of social norm compliance (Ruff et al., 2013). Interestingly, this is the same region that predicted individual differences in post-choice change in preference in non-social contexts (Jarcho et al., 2010; Qin et al., 2011; Mengarelli et al., 2013). Such a finding could be taken to suggest that the rejection of partisan support may be based on the same process of cognitive conflict that appears to determine alterations of preference and choice in non-political domains.

In light of this previous research, the aim of the current study was to provide evidence for a causal contribution of bilateral DLPFC in the alteration of political beliefs, and hence, partisan support. More specifically, it was hypothesized that activation of the DLPFC with a non-invasive brain stimulation would facilitate belief alteration if participants were exposed to a political campaign consistent with their initial belief during the stimulation. For example, those exposed to the Labor Party campaign were expected to display a change in initial political beliefs toward that of a more conservative, or right-wing (Conservative Party) orientation following transcranial stimulation of bilateral DLPFC. Therefore, despite ones initial political support, as well as the strength of their ideological position, the experience of cognitive dissonance and behavioral inhibition (initiated through enhanced DLPFC activity) was expected to result in an unfavorable impression of the observed campaign as reflected in strong support for the opposing party ideology.

To modulate the activity level of bilateral DLPFC, we delivered transcranial random noise stimulation (tRNS) bilaterally over these regions. It has been demonstrated that tRNS can modulate cortical excitability through the delivery of a randomly oscillating, bidirectional current (Terney et al., 2008). The particular importance of this technique is in its ability to avoid the homeostatic neural mechanisms, or hyper-polarization that have been observed during the repeated use of transcranial direct current (tDCS) techniques (Siebner et al., 2004; Stagg and Nitsche, 2011). In effect, any influence which tRNS may have on altering political beliefs will attest to its increasing potential in cognitive-neuroscience research in general. Further, any change in political beliefs which represent an opposing preference to that of the observed party campaign, could be taken as novel support for dissociable positive and negative regulatory networks within the later prefrontal cortex. That is, it may suggest a role for the DLPFC in the engagement of controlled processes implicated during the up-regulation of negative affect (Ochsner et al., 2004; Kaplan et al., 2007; Amodio et al., 2008).

## Methods

### Participants

Thirty-six Sussex University students (Female; *n* = 21, Male; *n* = 15) between the ages of 20–30 (mean = 24.6, *SD* ± 2.4) took part in the current study. All subjects presented written informed consent and were naive to both the purpose of the study, as well as the functions of transcranial random noise stimulation (tRNS). Participants were recruited through email and personal contact with the researcher and were excluded based on the criteria of neurological and psychiatric disorders (Poreisz et al., 2007; Rossi et al., 2009). All subjects used in this study were selected on the basis of social demographics, whereby they were required to have lived in Britain for a minimum of the past 3 years.

### Design

A 2X2 between-subjects design has been used, whereby two independent groups of participants participated in either an experimental condition involving the use of tRNS, or a control condition which involved a non-active, sham stimulation technique. Each stimulation group (experimental/sham) was further divided based on the political campaign video they were required to watch during the experiment. Therefore, the conditions used in the current study were as follows; tRNS + liberal, tRNS + conservative (experimental conditions), sham stimulation + liberal, and sham stimulation + conservative (control conditions). The change in political orientation from pre- to post-test has been calculated as the dependent variable.

### Transcranial random noise stimulation (tRNS)

The experimental condition involved applying tRNS over the bilateral DLPFC while subjects watched either a liberal or conservative party campaign. In the case of the control condition; non-active sham stimulation was applied while subjects watched one of the two campaign videos. The positions of the electrodes were the F3 and F4 positions of the international EEG 10–20 system (Fecteau et al., 2007a,b; Knoch et al., 2008; Keeser et al., 2011). The electrodes (7 × 5 cm) were covered with a sponge soaked with saline solution and stabilized using rubber bands. For the active tRNS condition, random noise current, driven by a battery–operated electrical stimulator (NeuroConn DC–Stimulator Plus) was gradually increased to a designated level of 2 mA at a sampling rate of 1280 samples per second (high frequency 101–640 Hz). The duration of stimulation was pre–programmed to 600 s using the CE approved stimulation device (DC-STIMULATOR PLUS; NeuroConn, Ilmenau, Germany). Transcranial stimulation was automatically terminated on reaching the required duration. The stimulation was also programmed to fade in/out over a duration of 20 s during at the beginning and end of the stimulation period.

**Figure 1:**
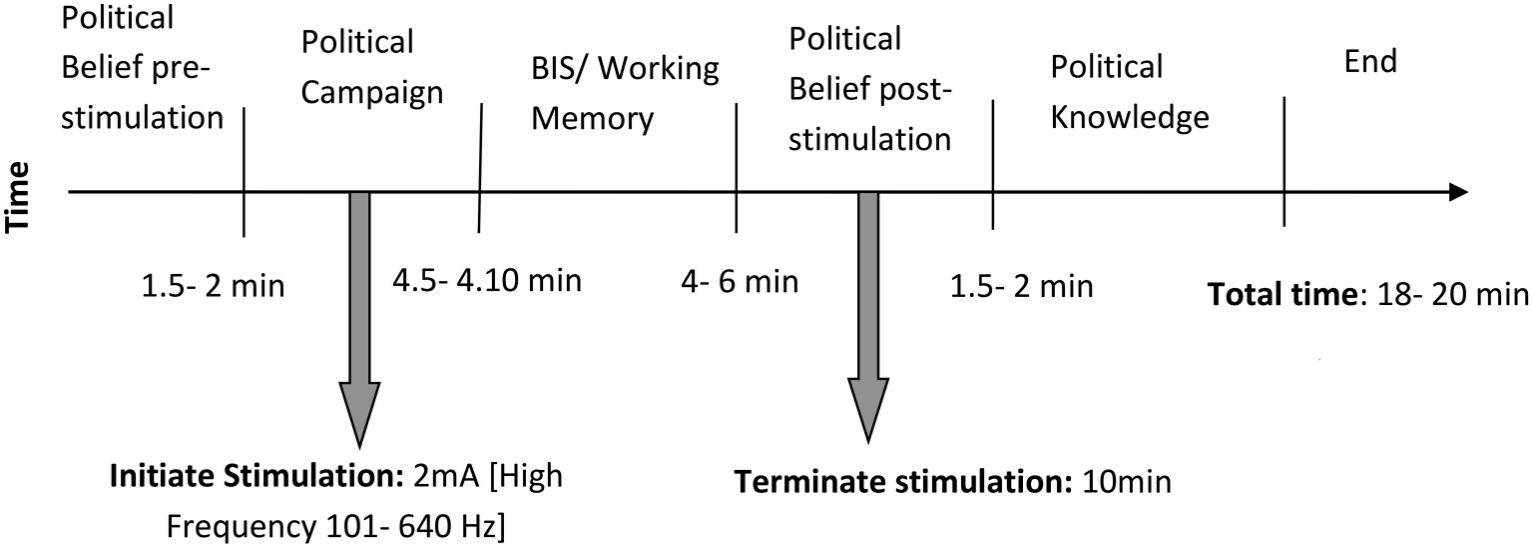
**Experimental Timeline (tRNS Stimulation)**.

tRNS stimulation began with the onset of the political campaign videos which lasted between 4.5 and 4.10 min in duration. Following the campaign video, participants required a further 6–8 min to complete the BIS, memory test, and post-test political attitude questionnaire (political knowledge was measured at the end of the study and was independent of stimulation–related changes in neural activity). tRNS has been shown to enhance cortical activity for an extended period of time beyond that of the actual stimulation procedure; 10 min tRNS induces an increase of MEP amplitude which is significant for up to 40 min post-stimulation (Laczó et al., 2014). As the entire experiment (beginning with the onset of stimulation) took approximately 12–15 min, the influence of DLPFC excitability was well within the period of tRNS after effects. Figure 1 (Experimental timeline) the control (sham) stimulation was accomplished by beginning the stimulation current in an identical manner to that of the active stimulation condition, but fading out after 20 s. Thus, participants perceived the initial tingling beneath the electrodes while receiving no active stimulation current for the rest of the experiment procedure. Recent research has demonstrated that study participants are generally unable to distinguish between active and non-active tRNS stimulation (Fertonani et al., 2011), suggesting that the absence of an active control site should not limit the reliability of results in the current study. Measures were also taken to ensure that no information about the stimulation procedure was communicated to participants prior to completion of the study.

### Procedure and analysis

Two pilot studies were carried out prior to experimentation. An independent group of 20 students were recruited to ensure that the political statements used in the current study were representative of a highly liberal or highly conservative ideology. The aim was to avoid any ambiguity with respect to particular political orientation being assessed. Conceptually ambiguous statements were removed leaving a total of 14 items per statement questionnaire. An additional 20 students were recruited to ensure that the questions referring to one's political knowledge were evenly divided into low, medium, and high levels of political knowledge. The knowledge questions to be used in the current study were subsequently based on the number of correct or incorrect answers provided by the sample of pilot study subjects. The research has been approved by the Sciences and Technology Cross-Schools Research Ethics Committee (C-REC) ethical process (ER/CC460/1), and was carried out in accordance with the British Psychological Society (BPS) code of conduct.

Participants were processed individually in a brain stimulation lab, whereby they were randomly assigned to a control or experimental condition. Twenty students (Female; *n* = 11, Male; *n* = 9) took part in the main stimulation experiment, with 16 subjects (Female; *n* = 10, Male; *n* = 6) assigned to the control condition. The conditions, however, were conducted by the same investigator, who was not blind with regard to the stimulation condition. Before beginning the experiment, all subjects were provided with an over-view of the study. The aim of the research was presented as a novel approach to investigating the neural basis of political beliefs. Each participant was further provided with a set of questionnaires (see Appendix A–D; 1–4) designed to measure political orientation prior to, and after viewing the allocated campaign advertisement (which was combined with tRNS or sham stimulation). The same questionnaires were administered to every participant. Participants were advised that the stimulation procedure would be applied as soon as they began answering the first set of political statement questions. The purpose of this was to prevent any bias in behavior change on beginning transcranial stimulation.

After reading the experimental instructions, and having them explained in detail by the experimenter, all subjects were asked to begin answering the first set of political attitude statements by coding one answer for each of the statements presented. Subjects were asked to relax and view the campaign video that followed, listening carefully to the argument put forward by the political candidate. Transcranial stimulation (active/sham) was initiated immediately with the onset of the political campaigns. Following the campaign videos, participants were required to answer questions referring to behavioral inhibition and their memory for the informational content covered by the political candidate. This was followed by the second set of political attitude statements and a political knowledge questionnaire. Participants were asked explicitly not to record their names on the questionnaires and were told that their answers would be immediately intermixed with those of the other participants. Participants were discouraged from selecting “socially acceptable” answers, and were advised to answer as honestly as possible throughout the study session.

In terms of Analysing political orientation; a score of 1, always represented a “highly liberal” attitude, whereas a score of 7 always represented a “highly conservative” attitude. Scores on each questionnaire were combined (after reverse scoring the items based on liberal-conservative direction) to provide a single measure of political orientation with higher numbers indicating a greater preference for Conservative, as opposed to Liberal political policies.

### Materials

Two political campaign videos were prepared, each of which was intended to portray a particular political orientation. Each of the videos was designed to reflect the most recent conservative (right–wing) or liberal (left–wing) policies proposed by the Conservative and Labor Parties. The topics which were covered in the campaign videos were designed to be as similar as possible, including topics relating to economic and political equality, collectivism/individualism and government control/free enterprise. The informational points were communicated in a similar sequence, with a similar length of time assigned to each topic. Therefore, both campaign videos were almost identical with respect to their total length, with 4.10 min allocated to the Labor Party (Ed Miliband) campaign and 4.05 min allocated to the conservative party (David Cameron) campaign. Both the recording quality and loudness of campaign videos were kept constant.

Two political attitude surveys were also designed to measure subject's political orientation before- and after-viewing the campaign video. Both attitude surveys consisted of a 14 item, additive Likert scale (Likert, 1932), designed to cover the main theoretical components of the liberal/-conservative political orientation. The 14 items on each survey were based as closely as possible on the campaign content, including statements referring to economic and political equality as well as collectivism and government control. All of the statements used in the current study were adapted from widely accepted political attitude surveys (e.g., World Values Survey, EES voter survey, European social survey, and British social attitudes survey, American National Election studies), attesting to the validity of the current liberal/-conservative attitude scale. Reliability estimates (see Results) further demonstrate that both liberal and conservative subscales maintain a standardized level of internal consistency, with all statement items appearing worthy of retention.

Each of the statements were phrased in an agree/disagree format, consisting of a seven-point Likert response scale (strongly agree, agree, slightly agree, neither agree nor disagree, slightly disagree, disagree, strongly disagree). In order to avoid the confounding influence of acquiescence effects, each set of items within a political attitude survey contained some question statements that were worded in one direction and some in the opposite direction (Schuman and Presser, 1981). Therefore, the selected items within each of the two surveys were balanced for question direction, with half the questions being worded in the direction of a highly liberal attitude and the other half worded in the direction of a highly conservative attitude (e.g., “*Taxes should be as low as possible even if welfare spending suffers,”* “*Income tax should be increased for people on higher than average incomes”*). In addition, the statement questions between the two surveys were matched as closely as possible with regard to particular attitude being assessed (e.g., “*Taxes and spending should be increased in order to improve health and education,” “Government should provide fewer services, even in health and education in order to reduce spending”*). Importantly, none of the statement questions were repeated at any stage in either of the attitude surveys. The purpose of this was to minimize retrieval of previously held beliefs.

A modified version of the behavioral inhibition system (BIS) was designed to cover four points relating to subjects current experience of anxiety, frustration, fear, and trustworthiness of the political campaign/-candidate they were exposed to (see Reliability Estimates).

To ensure that subjects paid attention to the campaign videos, we included a memory test for informational content. Seven questions were adopted from the main points covered by the candidates in each of the campaign speeches.

We also probed participant's political knowledge by administering a questionnaire consisting of 12 questions relating to current and past events in Britain, which were selected on the basis of number of correct responses displayed by participants in the pilot study. Four of these questions were consistently regarded as easy, four were found to be of medium difficulty, and four were found to be most difficult as determined by a similar student population.

## Results

### Reliability estimates

The scores for each political attitude survey were divided into either a liberal or conservative subscale. Reliability estimates (i.e., cronbachs alpha) demonstrate that both liberal (*a* = 0.67) and conservative (*a* = 0.78) political attitude subscales appeared to have good internal consistency, with all items appearing worthy of retention.

### Effects of tRNS on political attitude change

#### Political attitude changes after watching political campaign videos are summarized in Table 1 per group

A 2X2 ANOVA was carried out, with stimulation group (sham vs. tRNS) and campaign video (liberal vs. conservative) as the independent between-subject's variables. Total political attitude change from pre- to post-test was calculated as the dependent variable.

**Table 1:** **Means and SD for total political attitude change**.

**Stimulation**	**Campaign Group**	**Means**	***SD***	***N***
Control	Labor	1.88	1.64	8
	Conservative	0.00	4.17	8
tRNS	Labor	14.60	7.97	10
	Conservative	9.20	7.06	10

*SD, standard deviations; n, number of participants*.

Results indicate a significant main effect of stimulation group on subsequent change in political beliefs from pre- to post-test; *F*_(1, 29)_ = 25.14, *p* < 0.001, η^2^ = 0.2, with a higher mean belief change for tRNS stimulation conditions (*M* = 12.01) than that observed in the control stimulation conditions (*M* = 0.80). Therefore, the type of stimulation (tRNS or sham) appears to have a significant influence on changes in political orientation regardless of the political campaign participants had experienced.

However, when the type of stimulation was ignored, the political campaign video (liberal vs. conservative) had no significant main effect on political attitude change from pre- to post-test; *F*_(1, 29)_ = 2.54, *p* = 0.12, η^2^ = 0.02. When the influence of stimulation is removed, liberal, and conservative campaign groups appeared to score similarly on political attitudes; (Labor; *M* = 8.13; Conservative; *M* = 4.67), indicating that the particular campaign video that subjects were exposed to had no influence on subsequent alterations in political orientation.

In contrast to our expected hypothesis; results did not show any significant interaction between campaign group and stimulation type; *F*_(1, 29)_ = 0.30, *p* = 0.58, η^2^ = 0.2. This finding suggests that the effect of stimulation on political attitude change did not facilitate belief change as a result of watching the political campaigns *per se*. Those in the experimental (tRNS) groups appear to score higher on trait conservatism [higher scores represent conservative ideology/lower scores represent liberal ideology] regardless of whether they had been exposed to a Labor party or Conservative party campaign (Labor; *M* = 14.35; Conservative; *M* = 9.67). Those in the sham stimulation conditions exhibited little change in their political attitude from pre- to post-test.

These results demonstrate a significant increase in conservative political orientation following tRNS stimulation regardless of whether liberal or conservative campaign advertisements were viewed by participants.

A Three-way mixed ANOVA was also conducted in order to identify whether an interaction exists between stimulation group (sham vs. tRNS), campaign video (liberal vs. conservative) and political attitude scores from pre- to post-test. Stimulation group, campaign video, and time (pre vs. post) were used as the independent between/within-subject's variables. Mean political attitude change was calculated as the dependent variable.

**Figure 2:**
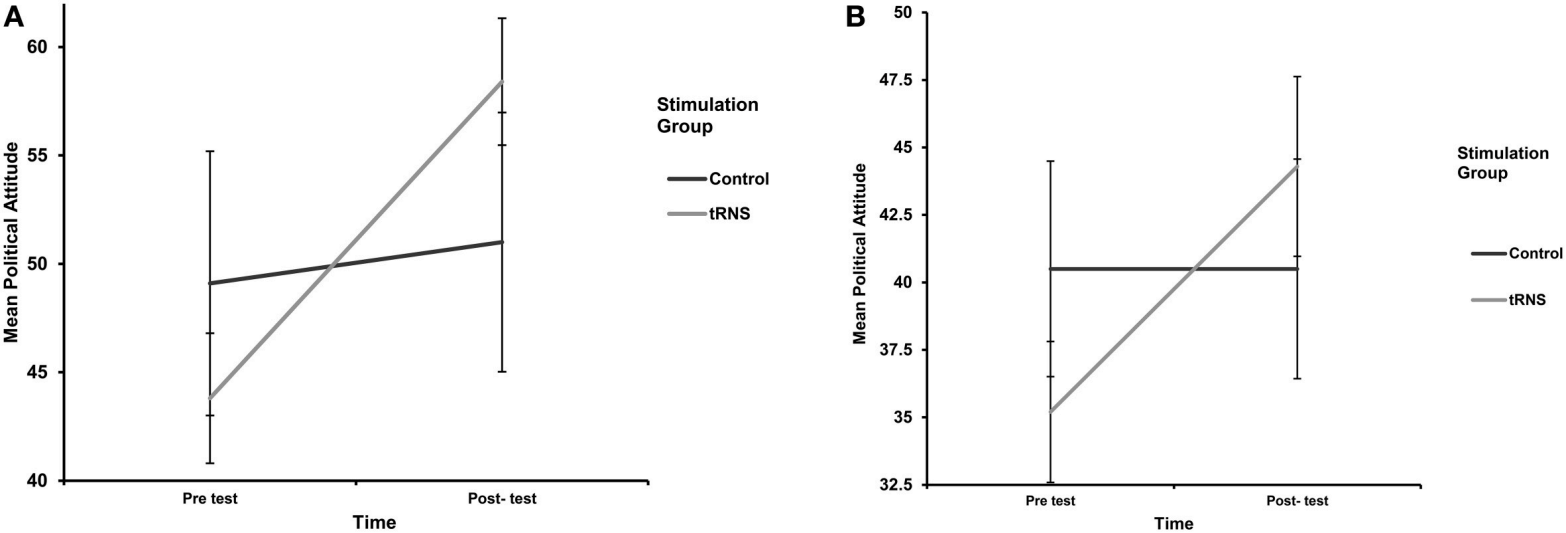
**(A)** Labor Party Campaign: mean political attitude. **(B)** Conservative Party Campaign: mean political attitude.

There was no significant interaction between stimulation type, campaign video, and pre–post political attitude scores; *F*_(1, 32)_ = 0.81, *p* = 0.37, $\eta_{p}^{2}=0.02$. There was also no significant interaction between campaign video (liberal vs. conservative) and political attitude scores from pre–post test; *F*_(1, 32)_ = 3.4, *p* = 0.08, $\eta_{p}^{2}=0.09$. Consistent with our previous results; the only significant interaction was found between stimulation type and pre–post political attitude change; *F*_(1, 32)_ = 29.5, *p* < 0.001, $\eta_{p}^{2}=0.48$ (Figure 2).

To ensure that these results were not driven by unexpected biases in the initial political orientation across the groups (see Table 2), we tested whether the initial political attitude interacted with political attitude change. First, a One-way ANOVA was conducted to test any baseline difference in initial political attitudes across each of the groups of subjects. The results indicated no significant difference between participants on initial political orientation *F*_(3, 32)_ = 2.4, *p* = 0.06, ω = 0.3. Given the uneven sample sizes, a non-parametric Spearman's test was used to determine the correlation between prior-political beliefs and total political attitude change. No significant correlation was found; *r* = −0.24, *p* (one-tailed) = 0.07. A one-tailed test was carried out as the original hypothesis suggested a change in conservative individuals toward that of a more liberal political orientation and vice versa.

**Table 2:** **Means and SD for political attitude: pre–post stimulation**.

**Stimulation**	**Campaign Group**	**Means**	***SD***	***N***
Pre-Control	Labor	49.13	17.22	8
	Conservative	44.25	8.56	8
Pre-tRNS	Labor	43.80	8.50	10
	Conservative	35.20	7.39	10
Post-Control	Labor	51.00	16.91	8
	Conservative	44.25	10.61	8
Post-tRNS	Labor	58.40	8.28	10
	Conservative	44.30	9.43	10

*SD, standard deviations, n, number of participants*.

**Figure 3:**
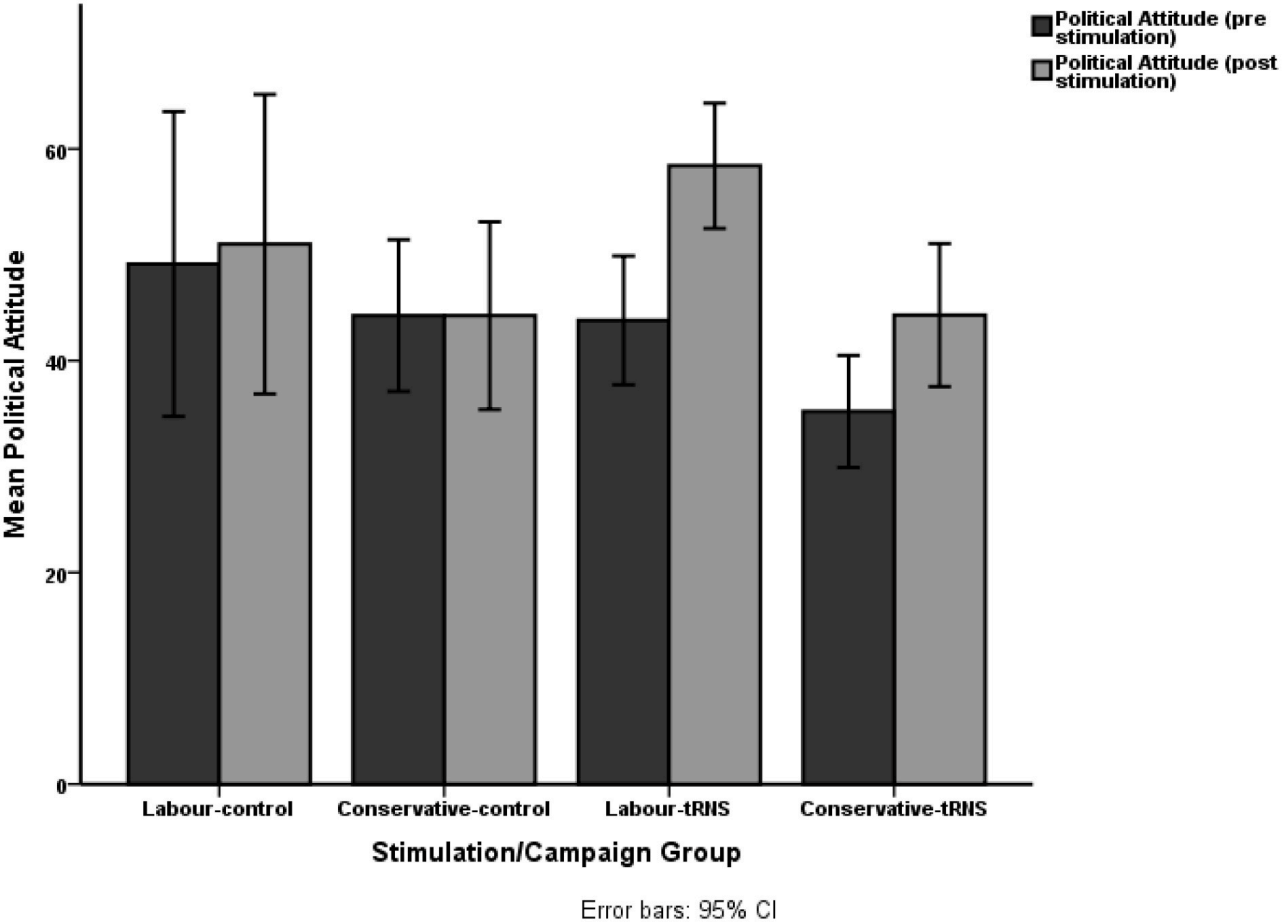
**Political attitude scores: Pre-post stimulation campaign**.

However, we also carried out a 2 (control/tRNS) X 2 (Labor/Conservative) ANOVA in order to determine whether the noticeably high *p*-value may have been affected by the campaign or stimulation groups that participants were assigned to. The results appear to indicate a significant main effect of campaign group (Labor/Conservative) on initial political orientation; *F*_(1, 32)_ = 5.13, *p* < 0.05, η^2^ = 0.004. Therefore, despite the non-significant interaction between initial political attitudes and post-test attitude change; we cannot ignore the findings that indicate significant differences in political beliefs ever before the experimental procedure had begun. More specifically, the Conservative campaign group appeared to demonstrate a lower level of conservative or right-wing ideology (*M* = 39.4, *SD* = 2.7) and the Labor campaign group demonstrated a higher rating on this scale (*M* = 46.5, *SD* = 2.7). The raw political attitude scores for pre- and post-stimulation are demonstrated in Figure 3.

### Interaction with auxiliary variables

We further examined whether the degree of political attitude change was moderated by other relevant auxiliary variables collected in this study (see Methods). However, we did not find any significant interaction between the degree of attitude change and other variables. First, political knowledge had no significant effect on belief change following tRNS stimulation; *F*_(1, 29)_ = 0.07, *p* = 0.78. This would suggest that, despite one's personal affiliation and strength of political support, enhanced activity of DLPFC has the potential to revoke pre-existing partisan support.

Second, the results showed no significant interaction between behavioral inhibition scores; *F*_(1, 29)_ = 0.99, *p* = 0.32, or between memory for informational content; *F*_(1, 29)_ = 0.88, *p* = 0.35 and subsequent change in political orientation. This contrasts with the role of DLPFC in cognitive dissonance reduction and avoidance-based motivation.

Finally, the change in political belief formation was independent of gender; *F*_(1, 29)_ = 0.50, *p* = 0.50, and age of participants (20–30 years); *F*_(1, 29)_ = 0.01, *p* = 0.90.

## Discussion

The current study aimed to specify a causal contribution of the dorsolateral prefrontal cortex (DLPFC) in altering political belief formation using a non-invasive brain stimulation technique (tRNS). The results did not support our original hypothesis that excitation of DLPFC with tRNS would facilitate alteration of political attitudes in response to watching campaign videos.

The findings of the current study indicate a crucial role of the DLPFC in political belief formation. However, the direction of this change was somewhat contradictory to the previous research. That is, the findings of the current study appear to only partly support those of Kato et al. (2009), which indicate the specific rejection of partisan support following enhanced activation of bilateral DLPFC.

Among the participants who were exposed to the Labor party (liberal) campaign, the change in subsequent political beliefs was in the expected direction, and represented a significant increase in conservatism or right-wing ideology. That is, despite the similarity in political statements, participants nonetheless placed their political preferences along a more conservative dimension following exposure to the Labor Party campaigns. From the outset, this finding appears to provide support for previous research indicating a specific role for the bilateral DLPFC in inductive (as opposed to deductive) reasoning (Goel and Dolan, 2004), thereby resulting in the rejection of support for the political candidate and party policies (e.g., Kato et al., 2009). However, if this change in political beliefs were a reflection of some kind of cognitive control or error feedback associated with the inconsistent intentions of the candidate/-campaign (e.g., Weissman et al., 2008; Burke et al., 2010), then we would also expect the Conservative Party campaign group to shown a similar rejection of the political ideology proposed. Surprisingly, however, this was not the case. Rather than rejecting conservative views, and altering beliefs accordingly (i.e., demonstrating increasing liberal ideology), enhanced DLPFC activity during the incorporation of Conservative Party campaigns actually led to a significant increase in conservative (as opposed to liberal) political beliefs. Contrary to our hypothesis, tRNS over DLPFC appeared to result in increased Conservative beliefs regardless of the political campaign subjects were exposed to. This finding would further indicate a null result in terms of the general up-regulation of negative affect associated with the right DLPFC (Ochsner et al., 2004; Kaplan et al., 2007; Amodio et al., 2008).

### Avoidance-regulation and conservatism

This particular pattern of results may be explained in terms of the specific psychological and neurological differences that underlie conservative and liberal belief systems (e.g., Jost et al., 2003a, 2007). As pointed out by Jost and Amodio (2012), it is limiting to suggest that two opposing political ideologies, which are based on advocating social change and rejecting inequality on the one hand, and maintaining tradition and acceptance of inequality on the other, could nonetheless depend on similar motivational processes. These highly opposing values must somehow originate in different cognitive mechanisms underlying motivation toward uncertainty and threat reduction.

If this is the case, then the enhanced cognitive dissonance and resulting avoidance motivation may not be associated with the observed candidate and party policies *per se*, but may reflect a general predisposition to avoid all uncertain and anxiety evoking situations which one may be exposed to. With this in mind, enhanced DLPFC may have resulted in an increased preference for security, certainty, and social dominance; traits which have been proposed as the defining characteristics of a conservative or right-wing ideology (Jost et al., 2003a,b, 2007; Weber and Federico, 2007; Federico et al., 2011). This is in contrast to traits of openness to social change and tolerance of uncertainty which are typically associated with a liberal belief system (e.g., Jost et al., 2004, 2008; Nosek et al., 2009). Such a proposal would lead us to suggest that the specific regulatory process (i.e., enhanced error feedback/cognitive conflict) evoked with transcranial stimulation may have biased the participants in favor of more conservative values and beliefs. Indeed, recent studies in political psychology have begun to document the differential regulatory mechanisms underlying right- and left-wing ideology (Janoff-Bulman et al., 2008, 2009; Janoff-Bulman, 2009), with recent fMRI investigations demonstrating a specific relationship between multi-dimensional scales of conservatism and activity of the right DLPFC (e.g., Zamboni et al., 2009). Zamboni and colleagues further discuss these findings in terms of a heightened sense of cognitive dissonance between self-interest and fairness that results in more complex social judgments in Conservative individuals.

### Avoidance-regulation and preference change

Despite the plausibility of a specific association between avoidance–based motivation and conservatism, it is necessary to point out the pre-existing characteristics of the sample groups used in the current study. Specifically, it is unrealistic to assume that the change in political orientation was devoid of prior preferences and beliefs. A particularly important finding that has been demonstrated in the current study is that of the null relationship or insignificant interaction between the campaign videos (Labor vs. Conservative) and transcranial stimulation. This would suggest that any change in political beliefs was exclusively the result of DLPFC activity and not necessarily due to the incorporation of the campaign information. Therefore, it remains important to acknowledge that despite the non-significant relationship between prior beliefs and changing political orientation, the subjects used in this particular study were nonetheless of a relatively liberal political orientation, with none of the participants scoring highly on initial conservative beliefs. Given the lack of any interaction between the campaign videos and that of DLPFC stimulation, the current findings, which represent a significant belief change in the direction of a conservative orientation, may actually conform to the expected results. Consistent with the research by Kato and colleagues, the significant alteration in political beliefs toward that of a conservative orientation could be taken to indicate a reduced affiliation of liberal individuals toward that of their initially supported political values.

If this proposal is taken to be true, then enhanced DLPFC activity could be taken to suggest a specific regulatory mechanism that underlies the rejection, as opposed to maintenance of political beliefs. Such a proposal would be in contrast to the assertion of avoidance–based regulation as a process specifically related to enhancing conservative beliefs. On the contrary, it would seem to suggest a specific role for the DLPFC in inducing a state of cognitive conflict toward existing political beliefs in general. This proposal would also be consistent with previous research on the neural basis of social conformity, whereby a prediction-error signal indicates the need to change one's opinion to meet that of normative group opinion (Klucharev et al., 2009, 2011).

However, recent research has begun to identify the role of a conflict mindset in reducing the tendency of people to over-estimate outgroup dissimilarity (Stern and Kleiman, 2015). That is, rather than enhancing in-group-outgroup differences; perceived distance is actually proposed to initiate a cognitive process of considering alternative perspectives (Kleiman and Hassin, 2013; Savary et al., 2015). This is of particular relevance to studies of political belief formation whereby people tend to view the attitudes of political outgroup members as being more different from their own attitudes than they actually are (Judd and Park, 1993; Robinson et al., 1995; Chambers et al., 2006; Graham et al., 2012; Westfall et al., 2015). Therefore, enhanced conflict monitoring and error-feedback processing could alternatively activate a more accurate representation of one's belief system, rather than changing or altering political beliefs *per se*.

### Limitations and future research

Given the current results, and the lack of any interaction between the campaign videos and cortical stimulation, we are left with little confirmatory evidence for the specific mechanisms through which DLPFC results in alteration of political beliefs. Future studies should be conducted with a broader population and greater sample of participants, thereby allowing for the inclusion of both conservative and liberal orientations at pre-test. This would inevitably shed light on the specific direction of belief change following enhanced activity of the dorsolateral prefrontal cortex. The small sample size used in the current study also poses a major limitation to the reliability of our results. Given that our participants were almost unanimously liberal in political orientation at pre-test, we must be cautious in drawing conclusions from this sample. The statistical analysis provided should be taken as support for future research, rather than in providing a definitive role for the DLPFC in political belief change.

The lack of any active control site further poses a limitation to the anatomical specificity of the DLPFC. With the current study, it is difficult to decipher as to whether the specific brain region being tested produces a behavioral affect that is significantly different to that of an active control. It is also possible that that the stimulation procedure may have induced discomfort in some participants, especially when applied over pre-frontal regions at higher stimulation intensities. Any unspecific behavioral effect could have had a substantial confounding effect on the reliability of results. Therefore, future research would benefit from the use of an active control site as opposed to a sham stimulation technique. This would allow for a reliable comparison in behavioral affects and the identification of a specific role for the DLPFC in belief change.

It is also necessary to take into account the way in which sensory information is represented in the brain by neurons. It is important to recognize that the final response given by the system is not based solely on the strength of the signal that codes for the target (i.e., rate of neuronal firing), but on the signal to noise ratio. That is, the ratio between the signal and other Information unrelated to the stimuli (neuronal noise). Miniussi et al. (2013) have argued that the “noise” induced by non-invasive brain stimulation can obstruct the synchronized ratio between the activity of neurons that code for the target and the activity of other neurons that are non-specific to the activity of the task. An uneven signal distribution caused by a sub-optimal level of “noise” is said to decrease the final performance as the response of neurons will not vary linearly with the characteristics of the target stimulus (Miniussi et al., 2013). With this in mind, it is possible that the tRNS induced noise could have enhanced trial variability, thereby resulting in attitude ratings “in the middle of the scale.” Given that the participants used in the current study were of a relatively liberal political orientation at pre-test, it would not be surprising that subsequent responses leaned toward a more pro-conservative orientation. If this is the case, then we would also expect participants who hold more conservative beliefs at pre-test to demonstrate a move toward a more liberal orientation. Unfortunately, cortical excitability alterations induced by tRNS are not yet fully understood and more research will be needed in this area in order to determine the influence of neuronal noise on behavioral trial by trial variability.

It is also important to mention the non-significant relation between political knowledge and subsequent change in beliefs. Given that political knowledge is taken as a measure of involvement; this finding would indicate the ability of DLPFC activity to over-ride any pre–potent emotional or personal response toward strongly held political beliefs. However, the social demographic background of the participants used in this study might pose limitations to its external validity. The particular strength of political orientation and emotional responses to current political events may be different to those held by other social and economic groups in society.

A further limitation of this study exists in that the two political attitude questionnaires were not counter-balanced prior to carrying out the study. Despite the reliability of the liberal– conservative scales being used, it would be beneficial for future studies to carry out a pilot test with an external sample of participants answering each questionnaire in a counter-balanced fashion. This would ensure that the questions being asked on each of the questionnaires are representative of the same political orientation. It would also be beneficial for future research to use more than one stimulus item (i.e., political campaign videos) as a measure of political orientation. Different measures focus more strongly on different components of attitudes, thereby allowing for ensure greater power and reliability in statistical results.

In addition, it would be of interest for future neuro–political research to look at the cerebral asymmetry of cognitive regulation. This differential influence of right and left prefrontal hemispheres might contribute to our understanding of the possible up-regulation of negative affect which might occur during partisan support, relative to the typical down-regulation of unwanted negative affect as seen in studies of implicit race bias toward out-groups (e.g., Cunningham et al., 2004).

The activation of both left and right hemispheres during Kato and colleagues study may have particular significance for altering political beliefs. Left frontal regions have been consistently associated with approach behavior, and are typically shown to be activated when participants rate others more favorably due to enhanced cognitive control over implicit negative emotions (Richeson et al., 2003; Cunningham et al., 2004; Wood et al., 2005). However, the additional involvement of right DLPFC may lend support to earlier accounts of the up-regulation of negative affect. That is, enhanced activation of right frontal regions have been demonstrated during the experience of withdrawal and anxiety, as well as in inhibitory behavior toward stimuli which are perceived as threatening to the individual (Sutton and Davidson, 1997; Dalton et al., 2005; Shackman et al., 2009). This proposal is consistent with neural findings which indicate that activation (as opposed to inhibition) of negative attitudes was associated with activity of the right DLPFC, with the degree of percentage change reflecting a correlation between trait and state anxiety measures (Wood et al., 2005). Neuroimaging research has further associated the right DLPFC with enhanced vigilance and sustained attention which have been proposed to underlie ones susceptibility to anxiety (Shackman et al., 2009). Such a proposal is consistent with this regions possible role in monitoring the behavioral representation of conflict as well as in controlling thought modification via error feedback. Although these findings are generally based on correlation, and do not necessarily address the issue of causation; they nonetheless lend support to an investigation of the bilateral DLPFC in political decision making, which may ultimately determine the relative degree to which political attitudes and prior beliefs are maintained or rejected.

### Unconscious rationalization

It is also interesting to note, that none of the participants in the current study reported any awareness of changes to their political beliefs, almost conclusively disagreeing with the possibility that political thoughts and values had been altered in any way. Therefore, during the conscious deliberation of political statements, it appears as though implicit cognitive control processes may have biased subsequent belief formation in the absence of conscious awareness. Although research has argued that rationalization and reappraisal must require some degree of conscious deliberation (Harmon-Jones and Harmon-Jones, 2002; Ochsner et al., 2002; Harmon-Jones et al., 2008; Van Veen et al., 2009; Jarcho et al., 2011); the findings of the current study would provide reason to speculate an unconscious role of the DLPFC in changing political orientation.

In support of this proposal, experimental investigations have indicated the possibility of dissociable brain components for executive control which appear to counter the notion that goal–directed behavior primarily requires conscious deliberation (e.g., Ochsner et al., 2002). Specifically, studies have indicated the presence of an operation goal programme on the one hand, with the conscious knowledge of its operation on the other (e.g., Frith et al., 2000). As such, it is possible that autonomously operated goals may guide cognition and behavior independent of conscious intention (Bargh and Ferguson, 2000; Chartrand and Bargh, 2002; Fitzsimons and Bargh, 2004; Bargh, 2005; Dijkstehuis et al., 2007; Ferguson et al., 2008). Such a proposal is consistent with recent research demonstrating that frontal lobe control regions of the brain are not necessarily essential for the generation of conscious volition (Koch and Tsuchiya, 2007).

## Conclusion

The findings of the current research question the reliability of current models of conscious control and deliberate political choice which currently dominant theories of human decision making in political science. Strongly held political beliefs appear to be surprisingly susceptible to alterations of neuronal regulatory processes, which have the potential to alter political belief systems as a function of inconsistent cognitive thought processes. The findings of the present study do not attempt to imply a deterministic or passive reflection of one's beliefs and attitudes; however, the ability of non-invasive stimulation to alter belief formation attests to the instability of political support and the powerful influence of uncertainty reduction in shaping societies ideological orientations.

## Funding

This work was supported by Japan Science and Technology Agency (JST).

### Conflict of interest statement

The authors declare that the research was conducted in the absence of any commercial or financial relationships that could be construed as a potential conflict of interest.
