# Supplementary material for: Alteration of Political Belief by Non-invasive Brain Stimulation
Source: Front Hum Neurosci. 2016 Jan 21;9:621. doi: 10.3389/fnhum.2015.00621 (PMC4720781; doi:10.3389/fnhum.2015.00621)
Supplement: Supplementary file 1 [file DataSheet1.DOCX]

***Appendix A.***

***Below are a number of statements about Britain today.***

***Please state how strongly you agree or disagree with each of them, using the following scoring code (Code only one answer for each statement).***

1. **Strongly Agree**
2. **Agree**
3. **Slightly Agree**
4. **Neither Agree nor Disagree**
5. **Slightly Disagree**
6. **Disagree**
7. **Strongly Disagree**

**__________________________________________________________________________________**

**Q1**. Government should increase taxes and spend much more on health and social services __**_**_

**Q2.** Rich people are being unfairly taxed ____

**Q3**. Private ownership of business and industry should be increased __**__**

**Q4.** Government should be the main provider of support for the unemployed ____

**Q5.** What is good for the most successful corporations, is ultimately good for all of us ____

**Q6**. Our society should do whatever is necessary to make sure that everyone has an equal opportunity to succeed __**_**_

**Q7**. Individual opportunity and freedom are greater when Government is smaller and less able to intervene in social and economic areas **_**___

**Q8**. It is more important for Government to help those according to their needs, rather than reward those for their hard work __**_**_

**Q9**. Government should redistribute income from the better off to those who are less well off ____

**Q10**. Government should provide fewer services even in health and education in order to reduce spending _**_**__

**Q11**. There is one rule for the rich and one rule for the poor **____**

**Q12**. The government should allow for the minimum income to rise more sharply than other income levels **___**

**Q13**. Social benefits and services in Britain make people lazy __**__**

**Q14**. Government regulation of business usually does more harm than good _**___**

***Appendix B.***

***Please state how strongly you agree or disagree with each of the following questions, using the following scoring code (Code only one answer for each statement).***

1. **Definitely Agree**
2. **Agree**
3. **Slightly Agree**
4. **Neither Agree nor Disagree**
5. **Slightly Disagree**
6. **Disagree**
7. **Definitely disagree**

**Q1**. I felt pretty anxious and on edge while watching the political campaign _____

**Q2**. I feel frustrated and annoyed after viewing this campaign _____

**Q3.** I feel worried about the ideas proposed by this candidate _____

**Q4**. I feel that this candidate is an untrustworthy person _____

***Each of the following questions is based on the video content which you have just watched. Please answered as many questions as possible.***

**Q1**. The current speaker is arguing for more, or less government?

**Q2.** What argument is made for the economic crisis at the beginning of the speech?

**Q3**. What makes life complicated for entrepreneurs?

**Q4**. Mention 1 question which this speaker poses to Ken Clarke and his business team.

**Q5**. Where will cuts be felt hardest?

**Q6**. Mention 1 key aim which is to be achieved in dealing with the nation’s national debt.

**Q7**. How much has the welfare bill increased over the past 10 years?

***Appendix C.***

***Below are a number of statements about Britain today.***

***Please state how strongly you agree or disagree with each of them, using the following scoring code (Code only one answer for each statement).***

1. **Strongly Agree**
2. **Agree**
3. **Slightly Agree**
4. **Neither Agree nor Disagree**
5. **Slightly Disagree**
6. **Disagree**
7. **Strongly Disagree**

**__________________________________________________________________________________**

**Q1**. Taxes should be as low as possible even if welfare spending suffers __**__**

**Q2**. Income tax should be increased for people on higher than average incomes ____

**Q3**. Nationalized industries are just as efficient as private industries _**__**_

**Q4**. Benefits for unemployed people are too high and discourage them from finding jobs ____

**Q5**. Big business benefits owners at the expense of workers **____**

**Q6**. The main reason that government has become bigger over the years is because it has gotten involved in things that people should do for themselves _**__**_

**Q7**. It is the Government’s responsibility to provide a job for everyone who wants one **____**

**Q8**. The Governments primary responsibility should be in providing industries with the support they need to grow ____

**Q9.** New welfare penalize those who work hard for their wealth rather than creating a fairer system ____

**Q10.** Taxes and spending should be increased in order to improve health and education ____

**Q11**. In Britain, the gap between those with large incomes and those with low incomes is too high **__**

**Q12**. Differences in income level encourage people to work hard and innovate ____

**Q13.** Those who are able to work and refuse the opportunity, should not expect societies support _**___**

**Q14**. Private enterprise is the best way to solve Britain’s economic problems ____

***Appendix D.***

***Below are a number of questions regarding general politics in Britain. Please answer as many questions as possible.***

**Q1**. Who is the current British Prime Minister?

**Q2**. In what building is the House of Commons located?

**Q3**. In what year was the last general election to the House of Commons?

**Q4**. Who is the current leader of the Liberal Democrats?

**Q5**. In which Middle Eastern country was the British embassy attacked by protesters in November 2011?

**Q6**. How many candidates can you choose when you vote in a general election?

**Q7**. Which is the more powerful house of parliament- The House of Lords or The House of Commons?

**Q8**. Which candidate competed with Ed. Miliband for leadership of the Labour Party in 2010?

**Q9**. Who is the current Chancellor of the Exchequer?

**Q10**. Who is the Shadow Chancellor of the Exchequer?

**Q11**. Who is the British Foreign Secretary?

**Q12**. What issue is likely to be put to vote in the next referendum (Britain)?

***Appendix 1.***

***Below are a number of statements about Britain today.***

***Please state how strongly you agree or disagree with each of them, using the following scoring code (Code only one answer for each statement).***

1. **Strongly Agree**
2. **Agree**
3. **Slightly Agree**
4. **Neither Agree nor Disagree**
5. **Slightly Disagree**
6. **Disagree**
7. **Strongly Disagree**

**__________________________________________________________________________________**

**Q1**. Government should increase taxes and spend much more on health and social services __**_**_

**Q2.** Rich people are being unfairly taxed ____

**Q3**. Private ownership of business and industry should be increased __**__**

**Q4.** Government should be the main provider of support for the unemployed ____

**Q5.** What is good for the most successful corporations, is ultimately good for all of us ____

**Q6**. Our society should do whatever is necessary to make sure that everyone has an equal opportunity to succeed __**_**_

**Q7**. Individual opportunity and freedom are greater when Government is smaller and less able to intervene in social and economic areas **_**___

**Q8**. It is more important for Government to help those according to their needs, rather than reward those for their hard work __**_**_

**Q9**. Government should redistribute income from the better off to those who are less well off ____

**Q10**. Government should provide fewer services even in health and education in order to reduce spending _**_**__

**Q11**. There is one rule for the rich and one rule for the poor **____**

**Q12**. The government should allow for the minimum income to rise more sharply than other income levels **___**

**Q13**. Social benefits and services in Britain make people lazy __**__**

**Q14**. Government regulation of business usually does more harm than good _**___**

***Appendix 2.***

***Please state how strongly you agree or disagree with each of the following questions, using the following scoring code (Code only one answer for each statement).***

1. **Definitely Agree**
2. **Agree**
3. **Slightly Agree**
4. **Neither Agree nor Disagree**
5. **Slightly Disagree**
6. **Disagree**
7. **Definitely disagree**

**Q1**. I felt pretty anxious and on edge while watching the political campaign _____

**Q2**. I feel frustrated and annoyed after viewing this campaign _____

**Q3.** I feel worried about the ideas proposed by this candidate _____

**Q4**. I feel that this candidate is an untrustworthy person _____

***Each of the following questions is based on the video content which you have just watched. Please answered as many questions as possible.***

**Q1**. According to this speaker, what should be the primary aim of welfare reform?

**Q2.** What argument is made against the current welfare system at the beginning of the speech?

**Q3**. What should be the primary responsibility of Government?

**Q4**. Mention one issue that should be taken into account before introducing “cuts”

**Q5**. What argument is made against the reform in cuts and spending?

**Q6**. Who is being penalized most with the introduction of welfare reform?

**Q7**. What is claimed to be responsible for the current economic crisis?

***Appendix 3.***

***Below are a number of statements about Britain today.***

***Please state how strongly you agree or disagree with each of them, using the following scoring code (Code only one answer for each statement).***

1. **Strongly Agree**
2. **Agree**
3. **Slightly Agree**
4. **Neither Agree nor Disagree**
5. **Slightly Disagree**
6. **Disagree**
7. **Strongly Disagree**

**__________________________________________________________________________________**

**Q1**. Taxes should be as low as possible even if welfare spending suffers __**__**

**Q2**. Income tax should be increased for people on higher than average incomes ____

**Q3**. Nationalized industries are just as efficient as private industries _**__**_

**Q4**. Benefits for unemployed people are too high and discourage them from finding jobs ____

**Q5**. Big business benefits owners at the expense of workers **____**

**Q6**. The main reason that government has become bigger over the years is because it has gotten involved in things that people should do for themselves _**__**_

**Q7**. It is the Government’s responsibility to provide a job for everyone who wants one **____**

**Q8**. The Governments primary responsibility should be in providing industries with the support they need to grow ____

**Q9.** New welfare penalize those who work hard for their wealth rather than creating a fairer system ____

**Q10.** Taxes and spending should be increased in order to improve health and education ____

**Q11**. In Britain, the gap between those with large incomes and those with low incomes is too high **__**

**Q12**. Differences in income level encourage people to work hard and innovate ____

**Q13.** Those who are able to work and refuse the opportunity, should not expect societies support _**___**

**Q14**. Private enterprise is the best way to solve Britain’s economic problems ____

***Appendix 4.***

***Below are a number of questions regarding general politics in Britain. Please answer as many questions as possible.***

**Q1**. Who is the current British Prime Minister?

**Q2**. In what building is the House of Commons located?

**Q3**. In what year was the last general election to the House of Commons?

**Q4**. Who is the current leader of the Liberal Democrats?

**Q5**. In which Middle Eastern country was the British embassy attacked by protesters in November 2011?

**Q6**. How many candidates can you choose when you vote in a general election?

**Q7**. Which is the more powerful house of parliament- The House of Lords or The House of Commons?

**Q8**. Which candidate competed with Ed. Miliband for leadership of the Labour Party in 2010?

**Q9**. Who is the current Chancellor of the Exchequer?

**Q10**. Who is the Shadow Chancellor of the Exchequer?

**Q11**. Who is the British Foreign Secretary?

**Q12**. What issue is likely to be put to vote in the next referendum (Britain)?
